# Lobbying by omission: what is known and unknown about harmful industry lobbyists in Australia

**DOI:** 10.1093/heapro/daad134

**Published:** 2023-10-21

**Authors:** Jennifer Lacy-Nichols, Shirae Christie, Katherine Cullerton

**Affiliations:** Centre for Health Policy, Melbourne School of Population and Global Health, The University of Melbourne, Level 5, 207 Bouverie St, Victoria 3010, Australia; Centre for Health Policy, Melbourne School of Population and Global Health, The University of Melbourne, Level 5, 207 Bouverie St, Carlton 3010 Victoria, Australia; School of Public Health, The University of Queensland, 266 Herston Rd, Herston 4006 Queensland, Australia

## Abstract

What is unknown about commercial lobbying is far greater than what is known. These omissions distort our understanding of the extent and nature of business influence on politics. Especially when businesses engage in practices that harm health, it is crucial for public health advocates to understand corporate lobbying to counter its influence. Our study proceeded in three phases. First, based on an international audit, we developed a list of the categories of information about lobbying that could be disclosed under four groups (lobby firms, lobbyists, organizations and activities) and benchmarked Australian lobbyist registers against this list. Second, we manually extracted data from lobbyist registers in eight jurisdictions, cleaned the data and created a relational model for analysis. Finally, we classified a sample of organizations as public health organizations or harmful industries to compare their activities. We identified 61 possible categories of information about lobbying in international lobbyist registers. When applied to Australian lobbyist registers, Queensland covered the widest range of categories (13, 21%), though many lacked detail and completeness. Australian lobbyist registers provided data on 462 third-party lobby firms across Australia, currently employing 1036 lobbyists and representing 4101 organizations. Several of these represented harmful industries, with gambling interests hiring the most third-party lobby firms. Ultimately, Australian lobbyist registers do not provide enough information to understand the full extent of lobbying activities taking place. Political transparency is important for public health actors to be able to monitor corporate political activity and to protect policy-making from vested interests.

**Contribution to Health Promotion**

- A first step to countering harmful influence is understanding it—this requires greater transparency of corporate lobbying.
- We develop a framework to evaluate government disclosure of lobbying, and show that Australian lobbyist registers do not provide sufficient information about who is trying to influence governments nor information about how and why different actors seek to influence governments.
- We offer suggestions to improve lobbying disclosures so it is easier for public health advocates to monitor corporate political activity.

## INTRODUCTION

Policy-making is fundamentally political. While public health evidence is one part of policy-making, policymakers also consider other values, interests, priorities and inputs (Hawkins and Parkhurst, 2016). In some cases, commercial interests are prioritized over public health. One example of this phenomenon is the slow and piecemeal adoption of the World Health Organization’s recommended Best Buy interventions to reduce the burden of non-communicable diseases, such as taxes, warning labels and restrictions on marketing tobacco, alcohol and sugary drinks (Allen *et al.*, 2022). These policies are designed to reduce the sale and consumption of harmful products. They also threaten the interests of powerful corporations.

To counter corporate profits being prioritized over public health, public health advocates require a greater understanding of *who* is trying to influence governments, *how* and *why*. Public health and political science research on corporate political activity has documented a range of strategies used to influence public decision-making (Ulucanlar *et al.*, 2016; Nyberg, 2021; Katic and Hillman, 2022). These include meeting with government officials, making campaign contributions, employing former government employees (referred to as the ‘revolving door’), writing policy submissions, participating in government committees, using the media to frame issues and debates and funding and establishing third-party organizations such as think tanks to promote business interests (often without a great deal of transparency). Lobbying and political advocacy are legitimate activities in democracies. However, the lobbying power of commercial actors and potential for governments to prioritize business interests over health, sustainability and human rights, have led many organizations to appeal for stricter regulations around corporate political activities (Access Info Europe *et al.*, 2022; International Institute for Democracy and Electoral Assistance, 2022).

Lobbying regulations and disclosure requirements vary substantially around the world. Transparency International defines lobbying as ‘any direct or indirect communication with public officials, political decision-makers or representatives for the purposes of influencing public decision-making, and carried out by or on behalf of a client or any organised group’ (Berg and Freund, 2015). One of the more common forms of disclosure is a lobbying register. The quality and completeness of these registers vary considerably. An analysis of 109 countries’ data governance and sharing practices found that few countries provided lobbying information in ways that enabled oversight and accountability (Global Data Barometer, 2022). Canada and Ireland are often regarded as countries with some of the best lobbying disclosure requirements (Huwyler and Martin, 2022; OECD, 2021). Both registers include consultant lobbyists (e.g. professional lobbyists employed by a third party) and in-house lobbyists (employed by a company, trade association or other advocacy group). In Ireland, each lobbyist report must detail the ‘relevant matter of the communications and the results they were intended to secure’—i.e. the purpose of lobbying (OECD, 2021).

In contrast, Australian disclosure requirements for lobbying require far less, with the federal lobbyist register described as a ‘tepid gesture towards transparency’ (Tham, 2019). The registers only apply to consultant lobbyists (who lobby on behalf of third-party clients). Not included in the register are lobbyists directly employed by companies, company executives and board members, non-governmental organizations, civil society organizations, not-for-profit organizations or associations, charities and foundations, think tanks, research centres, religious organizations and trade associations. The Australian federal register does not provide any information about the amount spent on lobbying or specific lobbying activities, such as when, where and with whom lobbyists met or the objective of the meeting or communication. Finally, most registers are effectively a snapshot in time, providing little historical information (or if they do, it is difficult to access and analyse).

The omission of information in government lobbyist registers risks misleading the public and policymakers about the extent and nature of corporate influence on public decision-making. Poor transparency may enable political decisions to favour business interests over public health. In 2022, Transparency International and the Open Government Partnership released their first Global Data Barometer, evaluating political transparency and disclosure (amongst other domains). For the lobbying domain, the report assessed the existence of data (e.g. available online), content of the data (e.g. information about money spent on lobbying), format of the data (e.g. unique identifiers used for lobbyists) and extent of the data (e.g. national coverage of the data) (Global Data Barometer, 2022). The report found that few governments provided information in ways that were easy to use, timely or complete, with only 19 of 109 countries providing lobbying data online (Global Data Barometer, 2022). This finding is consistent with decades of research and advocacy documenting the challenges of monitoring corporate lobbying (Gray and Lowery, 1996; Mialon, 2016; LaPira and Thomas, 2020; McKay and Wozniak, 2020; Lacy-Nichols and Cullerton, 2023).

To our knowledge, there has been only one extensive analysis of the makeup of lobbyist registers in Australia (Halpin and Warhurst, 2016). The study compared federal register data from 2012 and 2014, finding a gradual increase in registered firms, lobbyists and clients over time. In both years, around a quarter of all lobbyists and clients were concentrated in a small number of firms (though not always the same firms). Most firms were small scale, with a single lobbyist and single client. The study also classified clients into different groups (e.g. private company, national interest group, federal government, etc.), finding that around two-thirds of all clients were private companies. Other studies have also analysed the prevalence of the revolving door and ministerial diaries (which record meetings between ministers and lobbyists and other stakeholders) (Wood and Griffiths, 2018; Cullerton *et al.*, 2019; Robertson *et al.*, 2019; Lucas, 2021). Several reports have compared the evolution and differing requirements of federal, state and territory registers (Hogan, Murphy *et al.*, 2011, Hogan, Chari *et al.*, 2011; McKeown, 2014; Robertson *et al.*, 2018).

This study asks what information is shared—and not shared—about lobbying in Australia, and how this compares to international practice. We systematically analyse the information disclosed in Australian federal, state and territory lobbyist registers and compare this to international disclosure requirements. Our findings reveal numerous gaps and inconsistencies. In our discussion, we reflect on the utility of current lobbyist registers for analysing and monitoring corporate political practices and propose steps to improve the content and format of lobbyist registers in Australia and internationally.

## METHODS

This study proceeded in three phases. Phase 1 reviewed the online lobbyist registers in each jurisdiction and benchmarked the information provided against best practices internationally. Phases 2 and 3 were exploratory analyses of the data to see what patterns could be identified. Phase 2 included the extraction, cleaning and linking of data from each register to assess what information was provided and provide descriptive statistics on key metrics (e.g. count of lobby firms in each location). Phase 3 compared the lobbying activities of harmful industries and public health actors.

This study aimed to analyse information made publicly available by governments in lobbyist registers. For this reason, other sources of information about lobbyists and their connections—such as the biographies of elected officials (available on parliament websites), employment histories (via LinkedIn) and information about in-house lobbyists (via company websites and annual reports)—were outside the scope of this project. Opportunities to link with these data sources are explored in the discussion.

### Developing a framework to benchmark lobbying disclosures

Currently, there is no best practice standard against which to measure lobbying disclosures and transparency. For this study, we developed a list of variables that could be disclosed in a lobbying register. Our list is based on the OECD’s 2021 report *Lobbying in the 21st Century: Transparency, Integrity and Access* which included detailed Annexes documenting national lobbying standards, including definitions of lobbyists and lobbying activities, codes of conduct and disclosure requirements. A total of 21 countries and the EU had a lobbyist register or register of lobbyist meetings (Annex A.4). We documented the information each register made public and classified it as applying to lobby firms, lobbyists, clients or activities. Supplementary Appendix S1 presents our list of possible variables that could be disclosed against each category (also detailed in Table 1 of the results).

**Table 1:**
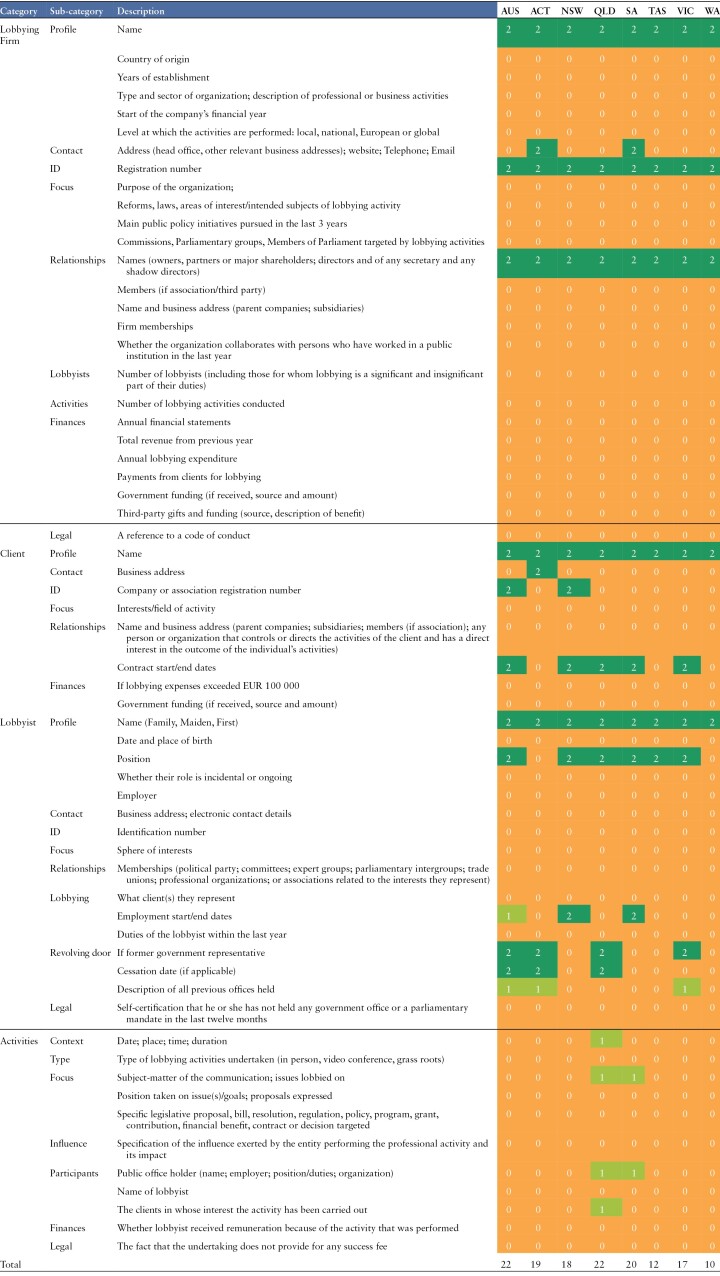
Benchmarking Australian lobbyist registers

*Note:* AUS: Australia; ACT: Australian Capital Territory; NSW: New South Wales; QLD: Queensland: SA: South Australia; TAS: Tasmania; VIC: Victoria; WA: Western Australia.

While the category ‘lobby firm’ is limited to professional lobby firms in Australia, international registers include a wider range of actors in that group. Rather than develop a specific classification for each type of actor, we have consolidated all the variables to present a simplified list. We note that some variables only apply to certain actors (for instance, payments from clients is relevant to consultant lobbyists and not all organizations will have members).

### Mapping data availability

We searched the websites of the federal, state and territory governments in Australia to identify publicly available lobbyist registers. All governments except the Northern territory (*n* = 8) had a register. Between 18 July and 7 August 2022, each register was reviewed to identify what information was provided about lobby firms, lobbyists and clients (see Supplementary Appendix S2). Once all variables were documented, we organized these into four overarching categories: lobby firms (organizations employing lobbyists), lobbyists (specific individuals listed in the registers), clients (predominantly commercial organizations who had hired lobby firms) and activities (e.g. meetings). These categories align with the organization of the lobbyist registers, and mostly corresponded with distinct actor groups (in a few cases, a lobby firm would be a client of another lobby firm). While some variables were present in all registers (e.g. the name of the client), others were only present in one or a few registers. This mapping is presented in Supplementary Appendix S2 and forms the framework used to guide data collection. Following this mapping, we finalized our list of variables (Supplementary Appendix S1), incorporating additional categories identified through this mapping exercise.

### Data collection and cleaning

Three data extraction templates were created in Excel based on the framework in Supplementary Appendix S1 for lobby firms, lobbyists and clients. As only two states provided information about lobbying activities (and South Australia only provided an annual summary), we did not collect data for this category.

Between 8 August and 19 September 2022, S.C. extracted data from each register and entered it into separate tables. The Federal and South Australian registers were available as downloadable .csv files. These were downloaded and the material was reorganized to fit into the templates. Data from the other registers were manually extracted. All data were entered as presented in an online register (i.e. no changes were made to capitalization, spelling or formatting at this stage).

Once data collection was complete, J.L.N. reviewed each table for completeness and accuracy. For each register, J.L.N. reviewed a sample of the largest firms (Barton Deakin, CMAX, GRACosway, Ogilvy, SEC Newgate), Pyne and Partners (present in all registers) and a random sample of five other large and small firms (see Supplementary Appendix S3 for a description of the largest lobby firms). A small number of minor typos in the names of some clients and lobbyists were identified. However, as these would be addressed during the cleaning and matching phase of analysis, they were not deemed a significant issue. While it is possible that there are errors in the data, we have done our best to confirm the validity of the data. We note that this is a static snapshot, and that the registers have been updated since data collection ended in September 2022. However, the findings presented here represent the best available information we have about the population of lobbyists and their clients in Australia to date.

Following this review, we consolidated the tables for each jurisdiction’s register into three master tables for lobby firms, lobbyists and clients. At this stage, there were 759 lobby firms, 1859 lobbyists and 7267 clients in our dataset. To identify the true count of unique lobby firms, lobbyists and clients, we took several steps to clean, simplify and match the data (see Supplementary Appendix S4 for details). Our aim was to match as many of the lobby firms, lobbyists and clients as feasible and to develop a single ‘defined name’ for each. We note that further cleaning and analysis are required to accurately match all the clients, however, this is beyond the scope of the current study due to its complexity and time-consuming nature. Because of this, our client counts are likely an overestimate of the true number of unique clients. Where possible we filtered our results to clients and lobbyists currently active according to the registers.

### Analysis

For phase 1 (benchmark Australian lobbyist registers), we used a simple three-point scale: 0 (did not have the variable), 1 (partially complete) and 2 (provided the variable). These results are presented in Table 1 of the results.

For phase 3, we compared the lobbying activities of harmful industries and public health organizations. To do this, we created a list of the largest tobacco, alcohol, ultra-processed food and gambling companies in Australia based on Euromonitor and IBISWorld market share. We used Excel’s FUZZY LOOKUP function to match this list to the client list. The Excel function helps to match textual data when the text is similar but not identical (e.g. ‘CocaCola’ and ‘The Coca-Cola Company’ or text with misspellings). The tool compares two sets of values, searches for the closest match and provides a similarity score for the match. We augmented this list by searching the client list for terms affiliated with third-party organizations (council, association, foundation, federation). We also identified six public health organizations in the lobbyist register (though it was not clear whether they hired the lobby firms, or if it was part of the firm’s pro bono work). These steps are detailed in Supplementary Appendix S4.

To analyse and visualize our findings, we built a relational data model using Power BI software. We linked the master tables via two categories that were in each table: the name of the lobby firm and the location of the lobbyist register (see Supplementary Appendix S4 for details). Our findings are presented below, with figures generated using Power BI Desktop.

## FINDINGS

### Phase 1. How complete are Australian lobbyist registers?

Compared to the list of possible variables disclosed internationally, Australian registers provided little information about the nature, extent and purpose of lobbying. Table 1 documents how each register performs against the range of possible variables used internationally. Based on our analysis, Queensland performs the best of the registers in terms of the range of variables provided (scoring equally with the federal register when quality of detail is considered), with Western Australia providing the least (noting that Northern territory does not have a register to evaluate).

While all registers required Australian Business Numbers (ABNs) for lobby firms, only two (Australia and New South Wales) required ABNs for clients. No register provided unique IDs for lobby firms, clients or lobbyists. This presents challenges for linking datasets, as we will discuss later. Other identifying information, such as the address of the lobby firm or client, was only provided in a few registers (Table 1).

Only four states provided information about a lobbyist’s previous government employment (Australia, Australian Capital Territory, Queensland and Victoria). Of these, only Australia, Australian Capital Territory and Queensland included had a specific entry for the ‘cessation date’ of the employment, a crucial piece of information for assuring compliance with ‘cooling off periods’ for former government representatives. Information about lobbying activities was lacking, with only South Australia and Queensland providing contact logs of lobbyist meetings. While these sometimes disclosed the topic of the meeting, this was often vague, for example, ‘introduction’.

### Phase 2. How many lobby firms, lobbyists and clients are there?

**Fig. 1:**
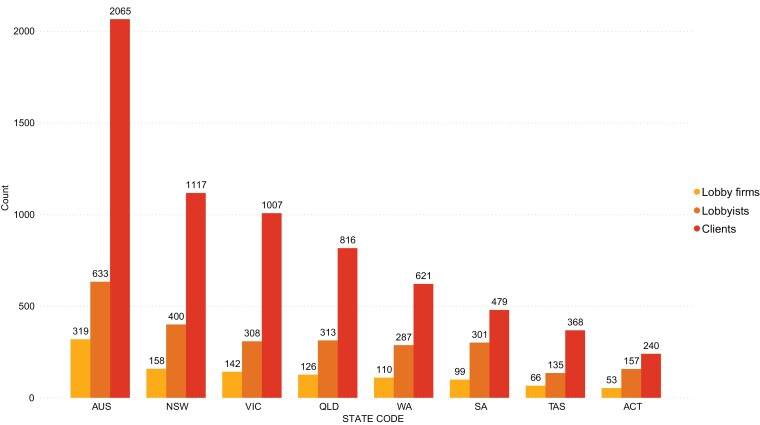
Count of lobby firms, clients and lobbyists by jurisdiction.

Based on the data we collected between August and October 2022, we estimate there were 462 lobby firms, currently employing 1036 lobbyists and representing 4101 clients across Australia (noting that this number is a snapshot in time). Figure 1 sets out the estimated number of lobby firms, lobbyists and clients in each jurisdiction. We note that while we have done our best to match duplicate lobby firms and lobbyists, the below figure overestimates the true number of clients, as it was beyond the scope of this study to manually review each individual client (*n* = 5808, including former clients) to ensure they were not the same company.

Several patterns can be observed from our analysis of the data. First, many of the same lobby firms, lobbyists and clients were present in more than one register, signalling those with the greatest breadth of political activity. In total, 25 (5%) lobby firms, 39 (4%) lobbyists and 50 (1%) clients were active in all eight jurisdictions. In contrast, 256 (55%) lobby firms, 528 (51%) lobbyists and 2887 (70%) clients were only active in one jurisdiction. Again, we note this figure may be slightly lower for clients, as some may be the same company but named differently in the registers.

Most lobby firms employed one lobbyist (*n* = 307, 66%), with 87 of these representing only one client. However, many firms had one lobbyist and many clients, with 44 single lobbyist firms representing ten or more clients, with the firm Media and Public Affairs Australia listing 73 clients. Many of the firms with the largest client rosters employed only a few lobbyists (see Supplementary Appendix S5). In the absence of detailed records of lobbying activities, it is not possible to measure how frequently each client engages in the services of a lobbying firm or the nature of the lobbying activities. For firms with more than one lobbyist, it is not possible to identify which lobbyists lobby on behalf of which clients.

Information about the revolving door (i.e. the movement between the public and private sectors) was only available in four registers. Based on disclosures, Victoria had the highest percent of lobbyists with a government background (47%), followed by Australian Capital Territory (46%), Australia (37%) and Queensland (27%). Three registers (Australia, Australian Capital Territory, Queensland) provided additional information about the date the lobbyist left the public sector (cessation date). The Australian and Queensland registers provided specific dates, whereas the Australian Capital Territory often listed only the month-year, or year.

The level of detail provided about former government employment varied significantly. For example, in the Victorian register, three generic categories were used, providing little information about the specific position, their portfolio or the dates of employment (e.g. ‘A CHIEF OF STAFF, SENIOR ADVISER OR ADVISER IN THE PRIVATE OFFICE OF A COMMONWEALTH OR STATE MINISTER, OR PARLIAMENTARY SECRETARY’). The Australian and Australian Capital Territory registers provided more information, however, the absence of a structured reporting framework meant that a total of 235 different categories were used. In some cases, more detailed information was provided (e.g. SENIOR POLICY ADVISOR TO FORMER PRIME MINISTER KEVIN RUDD MP), whereas in others general categories were used (e.g. ADVISER; ADVISOR; FEDERAL MINISTER; FORMER CHIEF OF STAFF; MINISTER; MINISTERIAL STAFF; SENIOR ADVISOR; STATE MINISTER). While some lobbyists disclosed several previous positions, most disclosed only one, preventing an analysis of the complete employment history and the various connections they might have within government or with other corporate actors. We also identified 96 lobbyists who were listed as both having and not having previous government employment. In those cases, we counted the lobbyist as having government experience, however, this inconsistency highlights the need for stricter oversight of lobbyist registers.

### Phase 3. Harmful industries vs public health actors

**Fig. 2:**
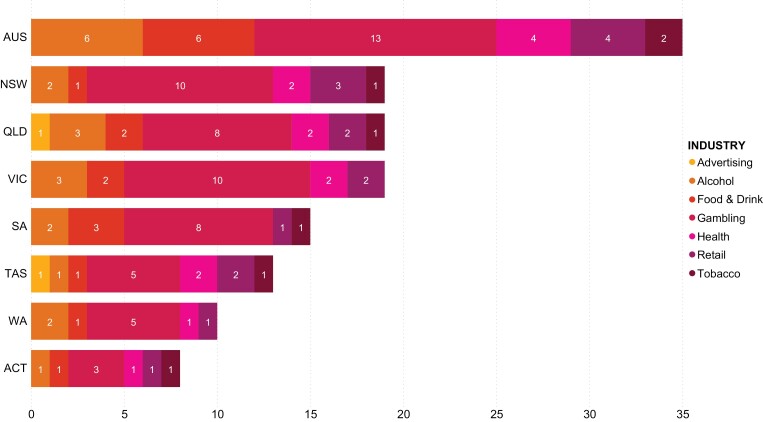
Lobbying breakdown by industry sector.

Finally, we analysed the lobby firms representing harmful industry and public health interests. Of the 222 harmful industry companies with the largest market share in Australia, only 22 appeared in the lobbyist registers at the time we collected data (see Supplementary Appendix S4 for details). The density of harmful industry lobbying varied by register, with most occurring in the federal register (Figure 2). While gambling clients were the most common across all jurisdictions, other harmful industries had a consistent, albeit smaller presence in most registers, with tobacco interests hiring lobby firms in all but two jurisdictions.

Our analysis identified three lobby firms that currently or previously represented both public health and harmful industry interests. As the registers do not provide a record of the activities of individual lobbyists, it was unclear what activities the lobbying firm performed for each client and whether the same lobbyist represented both public health and harmful industry interests. To explore this issue further, we reached out to the two public health organizations. One public health organization confirmed that they had hired the lobbying firm Ogilvy PR, but that the firm no longer represented the Australian Beverages Council in New South Wales (and that this relationship had ended before they began their contract). The other public health organization stated that they do not currently use any lobby firms. Of the three lobby firms previously engaged, they noted that Ogilvy PR was not engaged for lobbying, but rather for marketing advice regarding a fundraiser.

Following our consultation with both public health organizations, we again reviewed the registers to check whether the entries had been updated to reflect these changes. While some registers had been updated, others were not consistent with the information reported by the public health organizations.

## DISCUSSION

To our knowledge, this is the first study to quantify the number of registered lobby firms, lobbyists and clients in the Australian federal, state and territory registers. Our study updates Halpin and Warhurst’s (2016) analysis of the federal register in 2012 and 2014. We found similar patterns, with many single lobbyist/client firms, and a relatively small number of very large firms (Halpin and Warhurst, 2016). We note that these studies capture only a fraction of the total lobbying that occurs (the registers omit ‘in-house’ lobbyists directly employed by companies, for instance). Indeed, our finding that of the largest harmful industry companies in Australia, only a small number hired third-party lobby firms is likely a consequence of the limited dataset. Most companies likely have in-house lobbyists who are not required to be recorded in Australian registers.

Our overarching conclusion is that Australian lobbyist registers provided little information about lobbying. While Queensland provided the most information, the register only covered 13 of 61 possible variables. Information about lobbying activities (such as records of meetings and topics discussed) was conspicuously lacking. While Queensland and South Australia provided some information about lobbyists’ meetings, there was little detail about the purpose of the meeting. This information is crucial to understanding how commercial actors influence policy and whether they are successful.

Our study builds on and complements existing case studies on corporate political activity by providing an overview of the lobbying landscape. For instance, the corporate political activity taxonomy has been used to document the breadth of political practices in food, alcohol, tobacco and baby food industries (Savell *et al.*, 2014, 2016; Mialon *et al.*, 2016, 2021; Cetthakrikul *et al.*, 2021). Our study helps to contextualize the lobbying practices of specific companies amongst the political practices of other industry sectors. It also helps to provide an understanding of which actors are and aren’t engaging in lobbying (according to the disclosed data). Our study demonstrates the potential of using government datasets to analyse corporate political activity at scale. Increasingly, public health researchers are analysing large datasets, such as internal industry documents released as part of the litigation process or through freedom of information requests (Freudenberg, 2018; Maani Hessari *et al.*, 2019). Our study also provides a starting point for future monitoring efforts, for instance, tracking changes in the number of lobby firms, lobbyists and clients active in each jurisdiction, as well as the relationships between actors (Bennett *et al.*, 2023). This approach could be applied to other countries with public registers, including those with more detailed information about the types of lobbyists and their activities.

Our findings about the extent of the revolving door amongst lobbyists (i.e. the movement from government employment into the private sector) are almost certainly an underestimation. Indeed, a 2018 analysis from the Transparency Project run by the Guardian found that more than half of federal lobbyists in Australia have previously worked for government or major political parties (Knaus and Evershed, 2018). Previous public health studies have shown the importance of government experience and connections in helping to gain access to policymakers or influence policy-making (Williams, 2015; Scheffer *et al.*, 2020; Watts *et al.*, 2023). Efforts to map out the breadth of the revolving door across the lobbying sector can help to understand which companies may have better access to governments via the lobbyists they hire.

The challenges in assessing the revolving door are especially worthy of mention, as these raise questions about potential conflicts of interest if former government employees work in commercial sectors closely related to their previous role (and vice versa). One challenge arises from narrow disclosure requirements. We found only four jurisdictions required lobbyists to provide information about previous government employment. Moreover, in those registers, we found that most lobbyists provided only one government position, if any, which restricted our ability to understand the full extent of government experience a lobbyist may have. A second challenge for analysing the revolving door arises from the limitations in the legal instrument regulating lobbying in Australia. At the federal level, Australia only imposes an 18-month and 12-month restriction (for ministers/parliamentary secretaries and their staff, respectively) compared to Canada’s 5-year prohibition (McKeown, 2014). Australian lobbying regulation defines ‘lobbyist’ very narrowly, focussing solely on those who lobby on behalf of a third-party client. In practice, this means that Australia imposes a relatively short cooling-off period that applies only to a narrow range of lobbying positions (Wood and Griffiths, 2018). In Australia, over a quarter of former ministers and assistant ministers took roles with so-called ‘special interests’ (e.g. industry associations, lobby firms, consulting) after leaving office since 1990—positions technically allowed under the lenient regulations (Wood and Griffiths, 2018).

One strategy to address these challenges around the revolving door is to develop a more inclusive definition of ‘lobbyist’, for instance, including in-house lobbyists employed by companies, industry associations, consulting firms and not-for-profits (OECD, 2021). This would ensure that the restrictions on moving from government to private sector employment would apply to a much larger range of commercial actors and lobbying positions, which would also help to address some of the limitations with existing cooling-off periods noted above (Wood and Griffiths, 2018). A second opportunity to address the challenges of analysing the revolving door (in the absence of improved government regulations) is to augment our data with other datasets that provide more information about the employment history of lobbyists and public servants. Some of the sources used in previous studies include LinkedIn, Lexis-Nexis, LegiStorm and company websites (LaPira and Thomas, 2014). It is also important to advocate for improved lobbying regulations so that these data are routinely collected and shared publicly (Our Democracy, 2023).

Our finding that in some registers, public health organizations are listed as clients of lobby firms also representing harmful industry interests raises a number of observations and questions. Based on our engagement with two public health organizations, our first observation is that the registers are often not up to date (noting it is the responsibility of lobby firms to update information), nor do they provide clear information about the type or duration of services provided by the lobbying firm for client. This raises concerns about the completeness and credibility of the data in the registers. Indeed, a 2018 report from the Australian Auditor General found a ‘low level of compliance activity’ and a ‘lack of strategy around advice to Government representatives of their compliance monitoring responsibilities’ (Australian National Audit Office, 2018). We agree with calls from NGOs and researchers for greater transparency and disclosure of lobbying, and that the current lobbyist registers are not fit for purpose (Robertson *et al.*, 2018; Ng, 2020; Our Democracy, 2023).

Also concerning was the finding that lobby firms represented tobacco interests in six jurisdictions. This potentially contravenes Article 5.3 of the WHO Framework Convention on Tobacco Control which calls on governments to protect public health policies from the vested interests of the tobacco industry (World Health Organization, 2003). All governmental sectors are bound to comply with Article 5.3. While the type of service the lobbyist firms provide to the tobacco companies is unknown, the use of third-party lobbyists is a common strategy tobacco companies use to garner political support and influence public health policy (Watts *et al.*, 2023).

The potential for a lobbying firm to simultaneously represent clients with opposing interests raises wider questions about how conflicts of interest are identified and managed. One concern is that companies may share intelligence from the public health sector with their private sector clients. While this is not something we investigated (or found evidence of) in our study, there are examples where conflicts of interest have occurred. For instance, at the start of 2023, the multinational consulting firm PwC was found to have shared confidential government tax information with its private sector clients (Barrett, 2023). This highlights the potential risks of serving clients with conflicting interests and priorities. While many health organizations are taking steps to develop conflict of interest policies with their partners and funders, they may not think to do so for their other business relationships, for instance, external marketing teams, financial services or lawyers. Our findings suggest that it would be worthwhile for organizations to investigate what other clients these companies represent as part of a broader approach to conflicts of interest. We note that both public health organizations that we reached out to stated that they have policies and procedures in place to review potential relationships and conflicts of interest.

Lastly, in addition to the paucity of data, we found the design of the lobbyist registers makes it complex and time-consuming to access and analyse the data (likely one of the reasons why there are so few studies of the registers). Inconsistent spelling and labelling of lobby firms, lobbyists and clients made matching and comparing data across registers immensely difficult. Incorporating unique identifiers (that would match lobby firms, lobbyists and clients across registers) would be a tremendous aid. This has been implemented in databases built by NGOs and research groups, such as Open Secrets and LobbyView (both based in the USA) and Transparency International’s Integrity Watch initiatives in the EU, the UK, France, Italy and Chile (Kim and Kunisky, 2021; OpenSecrets.org., 2023; Transparency International EU, 2023). These databases also provide interactive dashboards and charts to allow easy searching for topics (e.g. top 10 lobbyists, or a breakdown of lobbying spending by industry sector). To our knowledge, no government register is so user-friendly.

Our study was limited to a specific set of harmful industry actors and specific time period (August–September 2022). Noting that companies may hire lobbyists for specific campaigns or policy issues, it is likely that a longer study would generate different results. Indeed, our review of the lobbyist registers in May 2023 following discussions with the public health organizations found that registers had been updated in the interim. Subsequent studies could analyse a wider breadth of actors and collect data over different time periods. A further option would be to automate elements of data collection, so that real-time data could be collected.

## CONCLUSION

Our study shows that Australian lobbyist registers do not provide sufficient information about who is trying to influence governments nor information about how and why different actors seek to influence governments. Nonetheless, our study highlights the opportunities for working within these constraints and using data analytics software to link disconnected datasets. Our empirical findings reveal which of the alcohol, tobacco, gambling and ultra-processed food companies have hired third-party lobby firms (and which have not). This can help public health advocates to develop a deeper understanding of the political strategies that harmful industries may use.

There are clear opportunities to improve political transparency and adopt approaches used internationally, such as the registers in Ireland and Canada that require more comprehensive disclosures of lobbying activities. There is a strong correlation between corporate political and financial influence and government inaction on public health policies (Boseley and McMahon, 2003; Fooks *et al.*, 2011; Miller *et al.*, 2011). To ensure that public health advocacy is effective, public health actors need to greatly expand their understanding of where, how, why and which commercial actors engage in politics.

## Supplementary Material

daad134_suppl_Supplementary_Appendixs_1

daad134_suppl_Supplementary_Appendixs_2

daad134_suppl_Supplementary_Appendixs_3

daad134_suppl_Supplementary_Appendixs_4

daad134_suppl_Supplementary_Appendixs_5

## Data Availability

The datasets analysed during the current study are available from the corresponding author upon reasonable request.
