# Supplementary material for: Lobbying by omission: what is known and unknown about harmful industry lobbyists in Australia
Source: Health Promot Int. 2023 Oct 21;38(5):daad134. doi: 10.1093/heapro/daad134 (PMC10590156; doi:10.1093/heapro/daad134)
Supplement: daad134_suppl_Supplementary_Appendixs_1 [file daad134_suppl_supplementary_appendixs_1.docx]

# Appendix 1. Information provided in lobbyist registers internationally

| **Category** | **Sub-category** | **Description** |
| --- | --- | --- |
| **Lobbying Firm*** | Profile | Name |
|  |  | Country of origin |
|  |  | Years of establishment |
|  |  | Type and sector of organisation; description of professional or business activities |
|  |  | Start of the company’s financial year |
|  |  | Level at which the activities are performed: local, national, European or global |
|  | Contact | Address (head office, other relevant business addresses); Website; Telephone; Email |
|  | ID | Registration number |
|  | Focus | Purpose of the organisation; |
|  |  | Reforms, laws, areas of interest/intended subjects of lobbying activity |
|  |  | Main public policy initiatives pursued in the last 3 years |
|  |  | Commissions, Parliamentary groups, Members of Parliament targeted by lobbying activities |
|  | Relationships | Names (Owners, partners or major shareholders; directors and of any secretary and any shadow directors) |
|  |  | Members (if association/third party) |
|  |  | Name and business address (parent companies; subsidiaries) |
|  |  | Firm memberships |
|  |  | Whether the organisation collaborates with persons who have worked in a public institution in the last year |
|  | Lobbyists | Number of lobbyists (including those for whom lobbying is a significant and insignificant part of their duties) |
|  | Activities | Number of lobbying activities conducted |
|  | Finances | Annual financial statements |
|  |  | Total revenue from previous year |
|  |  | Annual lobbying expenditure |
|  |  | Payments from clients for lobbying |
|  |  | Government funding (if received, source and amount) |
|  |  | Third party gifts and funding (source, description of benefit |
|  | Legal | A reference to a code of conduct |
| **Client*** | Profile | Name |
|  | Contact | business address |
|  | ID | Company or association registration number |
|  | Focus | Interests/field of activity |
|  | Relationships | Name and business address (parent companies; subsidiaries; members (if association); any person or organisation that controls or directs the activities of the client and has a direct interest in the outcome of the individual’s activities) |
|  |  | Contract start/end dates |
|  | Finances | If lobbying expenses exceeded EUR 100 000 |
|  |  | Government funding (if received, source and amount) |
| **Lobbyist** | Profile | Name (Family, Maiden, First) |
|  |  | Date and place of birth |
|  |  | Position |
|  |  | Whether their role is incidental or ongoing |
|  |  | Employer |
|  | Contact | Business address; Electronic contact details |
|  | ID | Identification number |
|  | Focus | Sphere of interests |
|  | Relationships | Memberships (Political party; committees; expert groups; Parliamentary intergroups; trade unions; professional organisations; or associations related to the interests they represent) |
|  | Lobbying | What client(s) they represent |
|  |  | Employment start/end dates |
|  |  | Duties of the lobbyist within the last year |
|  | Revolving Door | If former government representative |
|  |  | Cessation date (if applicable) |
|  |  | Description of all previous offices held |
|  | Legal | Self-certification that he or she has not held any government office or a parliamentary mandate in the last twelve months |
| **Activities** | Context | Date; Place; Time; Duration |
|  | Type | Type of lobbying activities undertaken (in person, video conference, grass roots) |
|  | Focus | Subject-matter of the communication; issues lobbied on |
|  |  | Position taken on issue(s)/goals; proposals expressed |
|  |  | Specific legislative proposal, bill, resolution, regulation, policy, program, grant, contribution, financial benefit, contract or decision targeted |
|  | Influence | Specification of the influence exerted by the entity performing the professional activity and its impact |
|  | Participants | Public office holder (name; employer; position/duties; organisation |
|  |  | Name of lobbyist |
|  |  | The clients in whose interest the activity has been carried out |
|  | Finances | Whether lobbyist received remuneration because of the activity that was performed |
|  | Legal | The fact that the undertaking does not provide for any success fee |

*For the purposes of this framework, the categories of Lobby Firm and Client include: consultant lobbyists, companies, NGOs/CSOs, charities and foundations, think tanks, research centres, religious organisations and trade associations
