# Supplementary material for: Lobbying by omission: what is known and unknown about harmful industry lobbyists in Australia
Source: Health Promot Int. 2023 Oct 21;38(5):daad134. doi: 10.1093/heapro/daad134 (PMC10590156; doi:10.1093/heapro/daad134)
Supplement: daad134_suppl_Supplementary_Appendixs_2 [file daad134_suppl_supplementary_appendixs_2.docx]

# Appendix 2. Variables present in Australian lobbyist registers

Lobbyist registers were reviewed between 18 July and 7 August 2022. This involved manually viewing multiple pages in each register to identify the full range of potential variables. As each register was formatted and organised differently, this was a time-consuming process. Further, there was significant variation in how the same variable was labelled in each register, for example, the name of the lobby firm was listed as the business name, business entity name, legal name, parent organisation, lobbyist business name, and name. In addition, not all lobby firms provided consistent information.

Table 1. Variables present in Australian government lobbyist registers

| **Category** | **AUS** | **ACT** | **NSW** | **QLD** | **SA** | **TAS** | **VIC** | **WA** |
| --- | --- | --- | --- | --- | --- | --- | --- | --- |
| **Lobby firms** |  |  |  |  |  |  |  |  |
| Business entity name | x | x | x | x | x | x | x | x |
| Trading name | x | x | x | x | x | x | x | x |
| ABN | x | x | x | x | x | x | x | x |
| Owner | x | x | x | x | x | x | x | x |
| Category |  | x |  |  |  |  |  |  |
| Date updated | x |  | x | x | x | x | x | x |
| Address |  | x |  |  |  |  |  |  |
| Suburb |  |  |  |  | x |  |  |  |
| State |  |  |  |  | x |  |  |  |
| Post code |  |  |  |  | x |  |  |  |
| Phone number |  | x |  |  |  |  |  |  |
| Email address |  | x |  |  |  |  |  |  |
| Website |  |  |  |  | x |  |  |  |
| **Lobbyists** |  |  |  |  |  |  |  |  |
| Name | x | x | x | x | x | x | x | x |
| Current position | x |  | x | x | x | x | x |  |
| Employee start date |  |  |  |  | x |  |  |  |
| Employee end date |  |  |  |  | x |  |  |  |
| Date added | x |  | x |  |  |  |  |  |
| Previous government employment (Y/N) | x |  |  | x |  |  |  |  |
| Former position | x | x |  |  |  |  | x |  |
| Cessation date | x | x |  | x |  |  |  |  |
| Associations |  |  |  | x |  |  |  |  |
| Employee restriction |  |  |  |  | x |  |  |  |
| Active |  |  | x |  |  |  |  |  |
| Employee type |  |  |  |  | x |  |  |  |
| **Clients** |  |  |  |  |  |  |  |  |
| Name | x | x | x | x | x | x | x | x |
| ABN | x |  | x |  |  |  |  |  |
| Address |  | x |  |  |  |  |  |  |
| Date added | x |  | x | x | x |  | x |  |
| Date removed |  |  | x | x | x |  | x |  |
| Active |  |  | x |  |  |  |  |  |
| Paid services |  |  |  | x |  |  |  |  |
| Foreign Principle |  |  | x |  |  |  |  |  |
| Country(s) |  |  | x |  |  |  |  |  |
| **Activities** | | | | | | | | |
| Lobby firm name |  |  |  | x | x |  |  |  |
| Lobby firm name ABN |  |  |  |  | x |  |  |  |
| Client |  |  |  | x | x |  |  |  |
| Date |  |  |  | x |  |  |  |  |
| Purpose |  |  |  | x |  |  |  |  |
| Government representative(s) |  |  |  | x |  |  |  |  |
| Annual summary of meetings (subject and public officials) |  |  |  |  | x |  |  |  |

*Note:* AUS: Australia; ACT: Australian Capital Territory; NSW: New South Wales; QLD: Queensland: SA: South Australia; TAS: Tasmania; VIC: Victoria; WA: Western Australia.
