# Supplementary material for: Lobbying by omission: what is known and unknown about harmful industry lobbyists in Australia
Source: Health Promot Int. 2023 Oct 21;38(5):daad134. doi: 10.1093/heapro/daad134 (PMC10590156; doi:10.1093/heapro/daad134)
Supplement: daad134_suppl_Supplementary_Appendixs_3 [file daad134_suppl_supplementary_appendixs_3.docx]

# Appendix 3: Lobby firm details

Table 1. Lobby firm details, information sourced from lobby firm websites 14 August 2023

| **Lobby firm name** | **Website** | **Affiliations** | **Services** | **Notes** |
| --- | --- | --- | --- | --- |
| Barton Deakin | https://bartondeakin.com/ | Liberal Party  National Party | Government Relations  Risk Management  Team Training | Founded in 2009 by a former leader of the New South Wales Liberal party.  Majority owned by WPP AUNZ, who also owns the Australian lobby firm HawkerBritton (affiliated with Labor Governments) |
| CMAX Advisory | https://cmaxadvisory.com.au/ | Bipartisan | Strategic Communications  Government relations  Corporate Strategy  Australian Weekly Report | Global company with offices in Canberra, Washington DC and London.  Partners include: G’day USA, Australian Chamber of Commerce, Fortem Australia, The Hands of Rescue, Soldier On and National Press Club of Australia |
| GRACosway | https://gracosway.com.au/ | Multi-partisan | Financial communications  Government relations and public policy advice  Corporate communications  Investor relations advice  Parliamentary and public inquiries counsel  Issues and crisis management  ESG advisory  Public affairs campaigning  Media and presentation training  Political and regulatory risk analysis  Management consulting | Part of the Clemender group, a holding company of advertising and marketing companies. The company also owns GCR Partners (NZ), GRA Partners (AU), and Porter Novelli (AU/NZ) – the latter two of which are also lobby firms in Australia. |
| Ogilvy | https://www.ogilvypr.com.au/ | Not described | Consumer PR and Influence  Health Communications & Public Affairs  Business Communications  Corporate Reputation  Social Media and influence  Investor and transaction communications  Environment, sustainability and corporate governance  Employee engagement  Public affairs and government relations  Brand identity and strategy  Trend reports and insights  Issues & Crisis Management | Owned by WPP AUNZ. Describe themselves as ‘a global creative communications agency’. Part of the Ogilvy Network, working across 93 countries. |
| SEC Newgate | https://www.secnewgate.com/ | Not described | Public Affairs  Reputation and crisis management  Research  ESG and Sustainability  Health Sciences and Wellbeing  Finance and Financial services  Manufacturing and Industry  Technology, Media & Telecommunication  Real Estate and Infrastructure  Consumer | 53 offices in 16 countries, a team of over 1200+ professionals |
| Pyne and Partners | https://pyneandpartners.com.au/ | Founded by former Minister in the Liberal/Coalition Governments | Strategic Communications  Strategic Advice  Public affairs  Government Engagement  Networking  Media Training and Support | The founder and managing director co-own the lobby firm GC Advisory, now Global & Corporate Advisory |
