# Supplementary material for: Lobbying by omission: what is known and unknown about harmful industry lobbyists in Australia
Source: Health Promot Int. 2023 Oct 21;38(5):daad134. doi: 10.1093/heapro/daad134 (PMC10590156; doi:10.1093/heapro/daad134)
Supplement: daad134_suppl_Supplementary_Appendixs_4 [file daad134_suppl_supplementary_appendixs_4.docx]

# Appendix 4: Steps for cleaning and matching Lobbyist register data

## preliminary Cleaning steps

Spreadsheets for each jurisdiction copied into three master sheets: lobby firms; clients; lobbyists

DATES cleaned: Federal and SA dates were in mm-dd-yyyy format; QLD dates were text and not recognised as date. Used Excel DATEVALUE and Text to Columns function to convert to dates and format as dd-mm-yyyy

ABNs: format as numbers and spaces removed (Find & Replace, Text to Columns function)

LOBBY FIRM, CLIENT AND LOBBYIST NAMES: Convert to uppercase (UPPER function); Remove leading and trailing spaces with TRIM(CLEAN(SUBSTITUTE(A1,CHAR(160)," "))) function

Use CONCAT function to create unique key for each lobby firm (combining BUSINESS ENTITY NAME and STATE CODE) and apply across lobby firm, lobbyist and client spreadsheets for matching.

## Lobby firms

We collected information for the below categories:

| **CATEGORY** | **ILLUSTRATIVE EXAMPLE (COMPILED FROM MULTIPLE REGISTERS)** |
| --- | --- |
| LOBBY FIRM DEFINED | HAWKER BRITTON |
| BUSINESS ENTITY NAME | HAWKER BRITTON GROUP PTY LIMITED |
| TRADING NAME | HAWKER BRITTON GROUP |
| ABN (AUSTRALIAN BUSINESS NUMBER) | 79109681405 |
| OWNER | SINGLETON OGILVY & MATHER (HOLDINGS) PTY LTD; STW MEDIA SERVICES PTY LIMITED |
| CATEGORY | COMPANY |
| DATE UPDATED | 19-Jun-22 |
| ADDRESS | Level 1, 2 King William Street |
| SUBURB | ADELAIDE |
| STATE | SOUTH AUSTRALIA |
| POST CODE | 5000 |
| PHONE NUMBER | (BLANK) |
| EMAIL ADDRESS | (BLANK) |
| WEBSITE | www.hawkerbritton.com |
| STATE CODE | SA |

**Original count of unique lobby firms: 759**

**Number of entries in spreadsheet: 1087**

Simplify LOBBY FIRM names with SUBSTITUTE function to remove unnecessary terms, punctuation and non-words (e.g., PTY, LTD, LIMITED, INC, CO, “()[].,-“ etc.); replace symbols with text (e.g., & to AND); remove text in parentheses e.g., “some text”; remove text following “trading as” or “T/A”; remove “THE” before “TRUSTEE”

Match lobby firm BUSINESS ENTITY NAMES (available for all entries) with TRADING NAMES. If multiple trading names, choose dominant match. For any uncertain matches, we confirmed with searches on Google, the lobby firm website and the Australian Business Register’s company/ABN lookup tool.

Power BI was used to review ABN matches across lobby firms. Four lobby firms were identified with matching ABNs (below table). However, these all occurred within the same register (NSW), and a review of the lobby firm websites and the Australian Business Register leads us to believe these were data entry errors. We have changed the ABNs in the spreadsheet accordingly (changed ABNs in BLUE below).

| **Business Name** | **Trading Name** | **Original ABN** | **Owners** | **STATE** | **Changed ABN** |
| --- | --- | --- | --- | --- | --- |
| NOTLEY - SMITH, BRUCE NEVILLE | LEYNOT ADVISORY | 38276001798 | BRUCE NOTLEY-SMITH | NSW | - |
| NOWRA CHEMICAL MANUFACTURERS PTY LTD | NOWRA CHEMICAL MANUFACTURERS PTY LTD | 38276001798 | FAY LAMONT; JOHN R LAMONT | NSW | 93001505988 |
| LIGHTHOUSE COMMUNICATIONS GROUP PTY LTD | LIGHTHOUSE COMMUNICATIONS GROUP | 84113421159 | PETER JOHN LAIDLAW | NSW | - |
| LUNIK | LUNIK | 83615178571 | EMILY MINSON; ZACKARY MCLENNAN | WA | - |
| LUNIK | LUNIK PTY LTD | 83615178571 | EMILY MINSON; ZACKARY MCLENNAN | TAS | - |
| LUNIK PTY LTD | LUNIK | 83615178571 | EMILY MINSON; ZACKARY MCLENNAN | AUS | - |
| LUNIK PTY LTD | LUNIK | 83615178571 | MR ZACKARY MCLENNAN; MS EMILY MINSON | QLD | - |
| LUNIK PTY LTD | LUNIK | 83615178571 | EMILY MINSONZACKARY MCLENNAN | SA | - |
| LUNIK PTY LTD | LUNIK | 83615178571 | ZACKARY MCLENNAN; EMILY MINSON | VIC | - |
| LUNIK PTY LTD | LUNIK | 84113421159 | EMILY MINSON; ZACKARY MCLENNAN | NSW | 83615178571 |

Several lobby firms were identified that listed different ABNs but linked to the same business website. A review of the lobby firm websites and owners led us to define these firms as the same company in practice. See table below for changes (highlighted in Yellow).

| **Original name** | **ABN** | **Matched name** |
| --- | --- | --- |
| CORNERSTONE GROUP AUSTRALIA | 99153936719 | CORNERSTONE GROUP AUSTRALIA |
| CORNERSTONE GOVERNMENT RELATIONS VIC PTY LTD | 73653641760 | CORNERSTONE GROUP AUSTRALIA |
| PREMIERNATIONAL PTY LTD | 71619450841 | PREMIERNATIONAL |
| PREMIER STATE CONSULTING PTY LTD | 26149964189 | PREMIERNATIONAL |
| 470 BOURKE PTY LTD. | 34639521681 | 470 BOURKE |
| EDWARD BOURKE | 94260862199 | 470 BOURKE |
| RICHARDSON COUTTS | 20126507211 | RICHARDSON COUTTS |
| FIPRA AUSTRALIA | 50078482596 | RICHARDSON COUTTS* |
| CT CORPORATE ADVISORY PTY LIMITED | 72600464855 | CT GROUP |
| CROSBY TEXTOR RESEARCH STRATEGIES RESULTS PTY LTD | 58101934454 | CT GROUP |
| MILNER STRATEGIC SERVICES | 24957388494 | GXO STRATEGIES |
| NEXT LEVEL STRATEGIC SERVICES PTY LTD | 27613857668 | GXO STRATEGIES** |

*(<https://fipra.com/network/fipra-australia/>)

**( As previously announced, Next Level is in the process of deregistering. For ongoing or future client matters please contact Cameron Milner at [---@gxostrategies.com.au](mailto:---@gxostrategies.com.au).)

Two firms were identified has having the same owners, however these were not changed on the spreadsheet as the websites remain distinct.

- COX INALL COMMUNICATIONS/DENTSU PUBLIC RELATIONS (Both owned by Dentsu, which lists 12 agencies as of 9 Jan 2023 on its website, including Cox Inall Communications, Cox Inall Change, Dentsu Creative and dentsu X).
- GRACOSWAY/GRA partners (GRACOSWAY owns GRA PARTNERS)– not yet changed

**Following these steps, we identified 462 unique lobby firms.**

## lobbyists

We collected information for the below categories:

| **CATEGORY** | **ILLUSTRATIVE EXAMPLE (COMPILED FROM MULTIPLE REGISTERS)** |
| --- | --- |
| DEFINED LOBBYIST NAME | ADAM HOWARD |
| INDIVIDUAL LOBBYIST NAME | ADAM HOWARD |
| CURRENT POSITION | MANAGING DIRECTOR |
| DATE ADDED | 12-NOVEMBER-2019 |
| EMPLOYEE START DATE | (BLANK) |
| EMPLOYEE END DATE | (BLANK) |
| ACTIVE | YES |
| LOBBY FIRM DEFINED | PYNE AND PARTNERS |
| LOBBY FIRM'S BUSINESS ENTITY NAME | PYNE AND PARTNERS PTY LTD |
| YES/NO? (FORMER GOVT. REPRESENTATIVE) | YES |
| FORMER POSITION OR SECTOR | CHIEF OF STAFF TO MINISTER FOR DEFENCE INDUSTRY |
| ASSOCIATIONS | (BLANK) |
| EMPLOYEE RESTRICTION | NONE |
| CESSATION DATE | 01-JULY-2018 |
| EMPLOYEE TYPE | PERSONS UNDERTAKING LOBBYING ACTIVITIES |
| STATE CODE | SA |

**Original count of unique lobbyists: 1859**

**Number of entries in spreadsheet: 3282 (three blank)**

To match lobbyists, the full list was copied, duplicates removed, and sorted alphabetically. We reviewed the entire list, first searching for all lobbyists with more than two names and common nicknames:

- three name text e.g., “JOHN ADAM SMITH” and search for “JOHN SMITH” or “JOHN ADAM”
- Nicknames e.g., BEN/BENJAMIN, BERNIE/BERNARD, BILL/WILLIAM

Compare names in lobbyist spreadsheet to see if work for same lobby firm – if so, we assumed they were the same individual. If so, replace all names with longest name (most unique). If the name was listed against more than one lobby firm, we conducted manual search to confirm if it was the same person (via LinkedIn, lobby firm websites and Google). In some cases, the name belonged to two individuals. In that case, the short version was used for one firm, and the long version used for another to ensure correct matching.

We used Power BI to identify lobbyists registered for more than one lobby firm (initial count n= 94). All names were searched to confirm if they were the same person via LinkedIn, Lobby firm website and Google. For individuals with the same name that are not the same person (n = 15 individuals), we modified the original name by adding “1” to the end of the last name to make distinct.

Use Power BI to match cleaned lobbyist name list with lobby firm list to create final count of lobbyists working for more than one firm (n = 64).

Of these, several lobbyists worked for firms that were strongly connected:

- 11 worked for GC ADVISORY and PYNE AND PARTNERS
- 2 worked for COX INALL COMMUNICATIONS and DENTSU PUBLIC RELATIONS

Although GRACOSWAY was listed as the owner of GRA PARTNERS, there were no lobbyists that are registered for both companies at the time of data collection.

**Following these steps, we identified 1420 unique lobbyists (including former lobbyists).**

## clients

We collected information for the below categories:

| **CATEGORY** | **ILLUSTRATIVE EXAMPLE (COMPILED FROM MULTIPLE REGISTERS)** |
| --- | --- |
| DEFINED CLIENT NAME | PHILIP MORRIS |
| CLIENT'S NAME ORIGINAL | PHILIP MORRIS LIMITED |
| CLIENT'S ABN | 65004694428 |
| CLIENT'S ADDRESS | (BLANK) |
| DATE ADDED | 25-March-2019 |
| DATE REMOVED | (BLANK) |
| FIRM DEFINED | CAPETAL ADVISORY |
| LOBBY FIRM'S BUSINESS ENTITY NAME | CAPETAL ADVISORY PTY LTD |
| LOBBY FIRM'S ABN | 86615732182 |
| FOREIGN PRINCIPAL | (BLANK) |
| ACTIVE | (BLANK) |
| COUNTRY(IES) | (BLANK) |
| PAID SERVICES ARE PROVIDED TO THIS CLIENT | (BLANK) |
| STATE CODE | AUS |

**Original count of unique clients: 7267**

**Number of entries in spreadsheet: 10,032 (69 blank)**

Simplify CLIENT FIRM names with SUBSTITUTE function to remove unnecessary terms, punctuation and non-words (e.g., PTY, LTD, LIMITED, INC, CO, “()[].,-“ etc.); replace symbols with text (e.g., & to AND);

Review text following “trading as”, “T/A” or “ATF” and search for matches to align names.

Remove “THE” at start of name (n = 226 entries)

The terms “ABN” and “ACN” were searched, and all entries providing this number were documented for future research and matching. Only one entry was identified with an ACN, which was its official name.

**Following the above steps, the overall list of clients was reduced to 5808 unique entries. However, it is highly likely that many of these are the same company and require further cleaning and analysis to match. Below, we suggest some steps for future project that are beyond the scope of this current project. In the results, we present client counts, but flag that these are likely over-estimations.**

Proposed future steps for additional cleaning and matching include:

- Search all state names and abbreviations for branches and consolidate.
- Use Power BI to match clients to ‘lobby firms defined’ and review client lists to see if similar client names (e.g. minor variations, misspellings, etc.). In some cases, clients list different names and ABNs, however the clients are likely linked (e.g., RAFAEL ADVANCED DEFENSE SYSTEMS and VARLEY RAFAEL AUSTRALIA – the former an Israeli private defense company, and the latter a joint venture of the same company in Australia). Matching similar entities like this could be useful for determining the percentage of domestic and foreign companies engaging lobbying services in Australia.

## Identifying harmful industry and public health actors

To create a list of relevant harmful industry actors, we searched Euromonitor market share data (2021) for:

- **Tobacco** (Cigars and Cigarillos; Cigarettes; Smoking Tobacco)
- **Alcohol** (Beer; RTDs; Wine; Spirits)
- **Ultra-processed foods** (Soft Drinks; Snacks)

And IBIS world 2021 data for:

- **Gambling** (Casinos; Lottery; Sports and racing betting; EGM)

We documented all companies listed in each dataset (excluding ‘Private Label’ and ‘Others’), including those with nil market share in 2021 (e.g., SABMiller Ltd) that were part of the dataset. This created a list of 222 unique potential ‘harmful industry’ companies.

We used Excel’s FUZZY LOOKUP function to search for these companies within the client list with a similarity threshold of 85 percent. 22 companies were identified (note the below table documents the category segment as well, so some companies are duplicated):

| **Industry** | **Category** | **Company Name** | **Client List** | **Similarity** |
| --- | --- | --- | --- | --- |
| Alcohol | Wine | Accolade Wines Ltd | ACCOLADE WINES | 0.9524 |
| Alcohol | Spirits | Accolade Wines Ltd | ACCOLADE WINES | 0.9524 |
| Alcohol | Beer | Asahi Group Holdings Ltd | ASAHI | 0.8818 |
| Alcohol | RTDs | Asahi Group Holdings Ltd | ASAHI | 0.8818 |
| Alcohol | Spirits | Asahi Group Holdings Ltd | ASAHI | 0.8818 |
| Alcohol | Wine | Australian Vintage Ltd | AUSTRALIAN VINTAGE | 0.9375 |
| Alcohol | RTDs | Beam Inc | BEAM | 0.9167 |
| Alcohol | Spirits | Beam Inc | BEAM | 0.9167 |
| Alcohol | Beer | Diageo Plc | DIAGEO | 0.9067 |
| Alcohol | RTDs | Diageo Plc | DIAGEO | 0.9067 |
| Alcohol | Spirits | Diageo Plc | DIAGEO | 0.9067 |
| Alcohol | Spirits | Mast-Jägermeister SE | JAGERMEISTER | 0.8809 |
| Alcohol | Wine | Treasury Wine Estates Ltd | TREASURY WINE ESTATES | 0.9655 |
| Food & Drink | Snacks | Green's General Foods Pty Ltd | GREEN'S GENERAL FOODS | 0.9394 |
| Food & Drink | Snacks | Mondelez International Inc | MONDELEZ | 0.8889 |
| Food & Drink | Snacks | Nestlé SA | NESTLE | 0.9077 |
| Food & Drink | Snacks | PepsiCo Inc | PEPSICO | 0.9231 |
| Food & Drink | Snacks | Unilever Group | UNILEVER | 0.9455 |
| Food & Drink | Soft Drinks | Asahi Group Holdings Ltd | ASAHI | 0.8818 |
| Food & Drink | Soft Drinks | Nestlé SA | NESTLE | 0.9077 |
| Food & Drink | Soft Drinks | PepsiCo Inc | PEPSICO | 0.9231 |
| Food & Drink | Soft Drinks | Red Bull GmbH | RED BULL GMBH | 1.0000 |
| Food & Drink | Soft Drinks | Unilever Group | UNILEVER | 0.9455 |
| Gambling | Casinos | Crown Resorts | CROWN RESORTS | 1.0000 |
| Gambling | Casinos | Delaware North | DELAWARE NORTH | 0.9803 |
| Gambling | Sports and racing betting | Entain PLC | ENTAIN | 0.9067 |
| Gambling | Casinos | Federal Group | FEDERAL GROUP | 1.0000 |
| Gambling | Sports and racing betting | PointsBet Australia | POINTSBET AUSTRALIA | 1.0000 |
| Gambling | Lottery | Tabcorp Holdings | TABCORP | 0.9231 |
| Gambling | Casinos | The Star Entertainment Group | THE STAR ENTERTAINMENT GROUP | 1.0000 |
| Tobacco | Cigars and Cigarillos | British American Tobacco Plc | BRITISH AMERICAN TOBACCO | 0.9517 |
| Tobacco | Cigarettes | British American Tobacco Plc | BRITISH AMERICAN TOBACCO | 0.9517 |
| Tobacco | Smoking Tobacco | British American Tobacco Plc | BRITISH AMERICAN TOBACCO | 0.9517 |
| Tobacco | Cigars and Cigarillos | Philip Morris International Inc | PHILIP MORRIS | 0.9200 |
| Tobacco | Cigarettes | Philip Morris International Inc | PHILIP MORRIS | 0.9200 |

We then searched the CLIENT LIST for terms indicating third party groups (alcohol, gambling, tobacco and ultra-processed food interests documented below). We also documented retailer and advertising organisations (e.g., Outdoor Media Association). We supplemented these searches with our own knowledge of relevant companies in these sectors.

Terms searched: **council** (n = 136); **association** (n = 261); **foundation** (n= 93); **federation** (n = 19)

Organisations identified (n = 25):

| **INDUSTRY** | **Category** | **CLIENT NAME FINAL** |
| --- | --- | --- |
| Advertising | Association | AUSTRALIAN ASSOCIATION OF NATIONAL ADVERTISERS |
| Advertising | Association | OUTDOOR MEDIA ASSOCIATION |
| Alcohol | Association | AUSTRALIAN DISTILLERS ASSOCIATION |
| Alcohol | Association | AUSTRALIAN LIQUOR STORES ASSOCIATION |
| Alcohol | Association | INDEPENDENT BREWERS ASSOCIATION |
| Alcohol | Association | SPIRITS AND COCKTAILS ASSOCIATION |
| Alcohol | Association | BREWERS ASSOCIATION OF AUSTRALIA |
| Alcohol | Association | VICTORIAN WINE INDUSTRY ASSOCIATION (WINE VICTORIA) |
| Alcohol | Association | WA NIGHTCLUBS ASSOCIATION |
| Food & Drink | Association | AUSTRALIAN BEVERAGES COUNCIL |
| Food & Drink | Association | AUSTRALIAN FOOD AND GROCERY COUNCIL |
| Food & Drink | Association | RESTAURANT AND CATERING INDUSTRY ASSOCIATION OF AUSTRALIA |
| Food & Drink | Association | AUSTRALIAN RETAILERS ASSOCIATION |
| Food & Drink | Association | INFANT NUTRITION COUNCIL |
| Gambling | Association | AUSTRALIAN HOTELS ASSOCIATION |
| Gambling | Association | AUSTRALIAN HOTELS ASSOCIATION NSW |
| Gambling | Association | AUSTRALIAN HOTELS ASSOCIATION SA |
| Gambling | Association | AUSTRALIAN LOTTERY AND NEWSAGENTS ASSOCIATION |
| Gambling | Association | CLUBS QUEENSLAND |
| Gambling | Association | COMMUNITY CLUBS VICTORIA |
| Gambling | Association | GAMING TECHNOLOGIES ASSOCIATION |
| Gambling | Association | NEW SOUTH WALES GREYHOUND BREEDERS OWNERS AND TRAINERS ASSOCIATION |
| Gambling | Association | RESPONSIBLE WAGERING AUSTRALIA |
| Retail | Association | AUSTRALASIAN ASSOCIATION OF CONVENIENCE STORES |
| Retail | Association | NATIONAL RETAIL ASSOCIATION |

We also identified public health organisations (n = 6):

| **Industry** | **Category** | **Public health organisation** |
| --- | --- | --- |
| Health | Association | CANCER COUNCIL AUSTRALIA |
| Health | Association | CANCER COUNCIL TASMANIA |
| Health | Association | DIETITIANS ASSOCIATION OF AUSTRALIA |
| Health | Association | PUBLIC HEALTH ASSOCIATION OF AUSTRALIA |
| Health | Association | ALCOHOL AND DRUG FOUNDATION |
| Health | Association | HEART FOUNDATION |

Our final list of 61 actors was classified into the below categories:

| **INDUSTRY** | **TYPE** | **CLIENT NAME FINAL** |
| --- | --- | --- |
| Advertising | Association | AUSTRALIAN ASSOCIATION OF NATIONAL ADVERTISERS |
| Advertising | Association | OUTDOOR MEDIA ASSOCIATION |
| Alcohol | Association | AUSTRALIAN DISTILLERS ASSOCIATION |
| Alcohol | Association | AUSTRALIAN LIQUOR STORES ASSOCIATION |
| Alcohol | Association | INDEPENDENT BREWERS ASSOCIATION |
| Alcohol | Association | SPIRITS AND COCKTAILS ASSOCIATION |
| Alcohol | Association | BREWERS ASSOCIATION OF AUSTRALIA |
| Alcohol | Association | VICTORIAN WINE INDUSTRY ASSOCIATION (WINE VICTORIA) |
| Alcohol | Association | WA NIGHTCLUBS ASSOCIATION |
| Alcohol | Company | ACCOLADE WINES |
| Alcohol | Company | ASAHI |
| Alcohol | Company | AUSTRALIAN VINTAGE |
| Alcohol | Company | BEAM |
| Alcohol | Company | DIAGEO |
| Alcohol | Company | JAGERMEISTER |
| Alcohol | Company | TREASURY WINE ESTATES |
| Food & Drink | Association | AUSTRALIAN BEVERAGES COUNCIL |
| Food & Drink | Association | AUSTRALIAN FOOD AND GROCERY COUNCIL |
| Food & Drink | Association | RESTAURANT AND CATERING INDUSTRY ASSOCIATION OF AUSTRALIA |
| Food & Drink | Association | AUSTRALIAN RETAILERS ASSOCIATION |
| Food & Drink | Association | INFANT NUTRITION COUNCIL |
| Food & Drink | Company | GREEN'S GENERAL FOODS |
| Food & Drink | Company | MONDELEZ |
| Food & Drink | Company | NESTLE |
| Food & Drink | Company | PEPSICO |
| Food & Drink | Company | UNILEVER |
| Food & Drink | Company | RED BULL GMBH |
| Gambling | Association | AUSTRALIAN HOTELS ASSOCIATION |
| Gambling | Association | AUSTRALIAN HOTELS ASSOCIATION NSW |
| Gambling | Association | AUSTRALIAN HOTELS ASSOCIATION SA |
| Gambling | Association | AUSTRALIAN LOTTERY AND NEWSAGENTS ASSOCIATION |
| Gambling | Association | CLUBS QUEENSLAND |
| Gambling | Association | COMMUNITY CLUBS VICTORIA |
| Gambling | Association | GAMING TECHNOLOGIES ASSOCIATION |
| Gambling | Association | NEW SOUTH WALES GREYHOUND BREEDERS OWNERS AND TRAINERS ASSOCIATION |
| Gambling | Association | RESPONSIBLE WAGERING AUSTRALIA |
| Gambling | Company | BET365 |
| Gambling | Company | CROWN RESORTS |
| Gambling | Company | DELAWARE NORTH |
| Gambling | Company | ECHO ENTERTAINMENT GROUP |
| Gambling | Company | ENTAIN |
| Gambling | Company | FEDERAL GROUP |
| Gambling | Company | LOTTOLAND AUSTRALIA |
| Gambling | Company | POINTSBET AUSTRALIA |
| Gambling | Company | SKYCITY CASINO |
| Gambling | Company | SPORTSBET |
| Gambling | Company | STAR ENTERTAINMENT GROUP |
| Gambling | Company | TABCORP |
| Health | Association | CANCER COUNCIL AUSTRALIA |
| Health | Association | CANCER COUNCIL TASMANIA |
| Health | Association | DIETITIANS ASSOCIATION OF AUSTRALIA |
| Health | Association | PUBLIC HEALTH ASSOCIATION OF AUSTRALIA |
| Health | Association | ALCOHOL AND DRUG FOUNDATION |
| Health | Association | HEART FOUNDATION |
| Retail | Association | AUSTRALASIAN ASSOCIATION OF CONVENIENCE STORES |
| Retail | Association | NATIONAL RETAIL ASSOCIATION |
| Retail | Company | COLES |
| Retail | Company | METCASH |
| Retail | Company | WOOLWORTHS |
| Tobacco | Company | BRITISH AMERICAN TOBACCO |
| Tobacco | Company | PHILIP MORRIS |

## Data analysis

To analyse and visualise our findings, we built a relational data model using Power BI software. We linked the tables via two properties that were in each of the tables: the name of the lobby firm (LOBBY FIRM DEFINED) and the location of the lobbyist register (STATE CODE). Because we had cleaned the data and identified matching lobby firms, lobbyists and clients, this allows us to generate unique count for lobby firms, lobbyists and clients (i.e., avoiding double counting a lobbyist or client that was listed in multiple register for the same lobby firm). This allowed us to compare the total number of unique lobbyists and clients linked to each lobby firm. Because each entry in our tables was linked to a specific location, we could also count in how many locations each lobby firm, lobbyist and client was registered.
