# Supplementary material for: Lobbying by omission: what is known and unknown about harmful industry lobbyists in Australia
Source: Health Promot Int. 2023 Oct 21;38(5):daad134. doi: 10.1093/heapro/daad134 (PMC10590156; doi:10.1093/heapro/daad134)

# Appendix 5. Comparison of the number of clients and lobbyists by firm

Note that this is the aggregate number across all jurisdictions. Not all lobbyists and clients will be active in each location, so the ratio may vary depending on the jurisdiction.

Figure 1. Comparison ranked by number of clients (top 30)


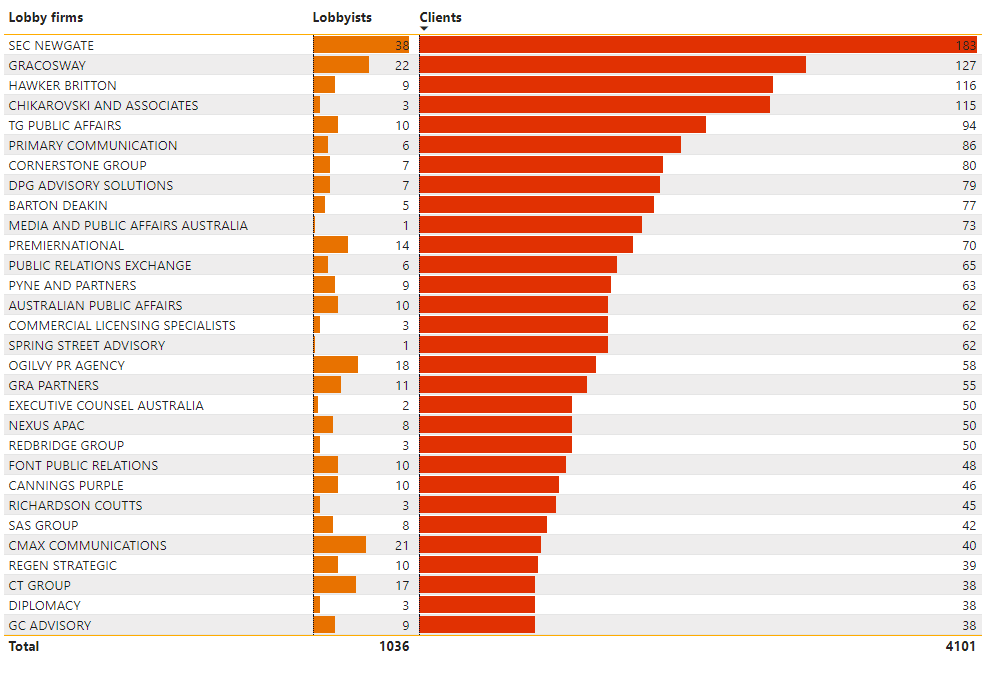


Figure 2. Comparison ranked by number of lobbyists (top 30)


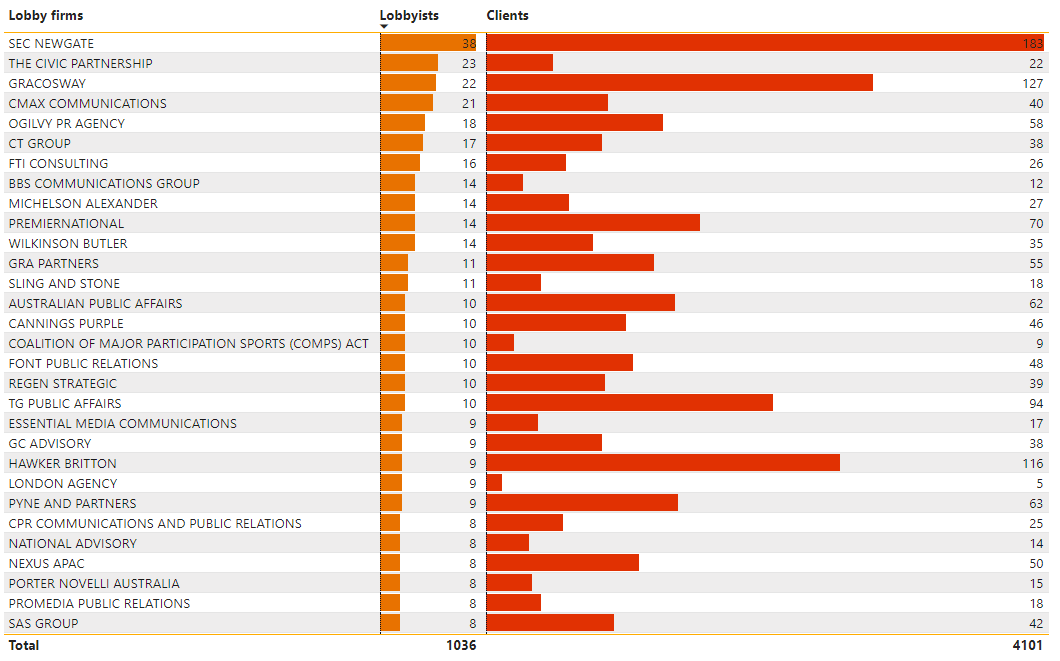

Supplement: daad134_suppl_Supplementary_Appendixs_5 [file daad134_suppl_supplementary_appendixs_5.docx]
